# The Protective Effect of Alpha-Lipoic Acid in Lipopolysaccharide-Induced Acute Lung Injury Is Mediated by Heme Oxygenase-1

**DOI:** 10.1155/2013/590363

**Published:** 2013-03-19

**Authors:** Yu-Chieh Lin, Yuan-Shu Lai, Tz-Chong Chou

**Affiliations:** ^1^Graduate Institute of Life Sciences, National Defense Medical Center, Taipei, Taiwan; ^2^Department of Chest, Taipei City Hospital, Taipei, Taiwan; ^3^Department of Biomedical Engineering, National Defense Medical Center, Taipei, Taiwan

## Abstract

Alpha-lipoic acid (ALA), occurring naturally in human food, is known to possess antioxidative and anti-inflammatory activities. Induction of heme oxygenase-1 (HO-1) has been reported to exhibit a therapeutic effect in several inflammatory diseases. The aim of study was to test the hypothesis that the protection of ALA against lipopolysaccharide-(LPS-) induced acute lung injury (ALI) is mediated by HO-1. Pre- or posttreatment with ALA significantly inhibited LPS-induced histological alterations of ALI, lung tissue edema, and production of proinflammatory cytokine, cytokine inducible neutrophil chemoattractant-3, and nitrite/nitrate in bronchoalveolar lavage fluid. In addition, the inflammatory responses including elevation of superoxide formation, myeloperoxidase activity, polymorphonuclear neutrophils infiltration, nitrotyrosine, inducible nitric oxide synthase expression and nuclear factor-kappa B (NF-**κ**B) activation in lung tissues of LPS-instilled rats were also markedly reduced by ALA. Interestingly, treatment with ALA significantly increased nuclear factor-erythroid 2-related factor 2 (Nrf2) activation and HO-1 expression in lungs of ALI. However, blocking HO-1 activity by tin protoporphyrin IX (SnPP), an HO-1 inhibitor, markedly abolished these beneficial effects of ALA in LPS-induced ALI. These results suggest that the protection mechanism of ALA may be through HO-1 induction and in turn suppressing NF-**κ**B-mediated inflammatory responses.

## 1. Introduction

Acute lung injury (ALI), a frequent critical complication in patients with sepsis or infection, has a high mortality rate of 30% to 40% despite advanced technique and support treatment used [1]. So far, no specific and effective treatment for ALI has been established. ALI is characterized by damage to the alveolar-capillary system and increased pulmonary vascular permeability, thereby resulting in pulmonary edema, hypoxia, and polymorphonuclear neutrophils (PMNs) infiltration into the alveolar space, which finally impairs respiratory function [2]. A sustained and uncontrolled pulmonary inflammation has been believed to play an important role in the pathogenesis of ALI. Thus, suppression of innate immune system-mediated inflammatory responses may be a potential strategy to attenuate the progression of ALI. Lipopolysaccharide (LPS), the major component of the utter membrane of Gram-negative bacteria, has been considered a key molecule triggering innate immunity and acute inflammatory responses through overproduction of proinflammatory cytokines, chemokines, reactive oxygen species (ROS), and nitric oxide (NO), and in turn resulting in ALI [3, 4]. Although there is no ideal animal model for ALI, the symptoms of rats with LPS-induced ALI have close resemblance to the pathological changes observed in human [5]. Moreover, intratracheal administration of LPS into lungs may be an ideal experimental model of ALI, for it causes lung injury without resulting in systemic inflammation and multiorgan failure [6].

Heme oxygenase (HO), a stress-responsive protein that can be induced by several inflammatory stimulates, degrades heme into three products: biliverdin, free Fe^2+^ irons, and carbon monoxide (CO) [7]. Three mammalian HO isoforms (HO-1, HO-2, and HO-3) have been identified. The HO-1 gene is inducible, whereas the genes encoding HO-2 and HO-3 are constitutively expressed. The transcription factor nuclear factor-erythroid-2-related-factor-2-(Nrf2-) mediated HO-1 induction has been demonstrated to exert a protective effect against the cytotoxicity of oxidative stress, inflammation, and apoptosis [8]. A previous study has indicated that activation of HO-1 ameliorates the injury in LPS-induced ALI in rats [9]. These findings suggest that agents with HO-1 activating effect agents may have a protective effect on the ALI induced by LPS. 

**Figure 1:**
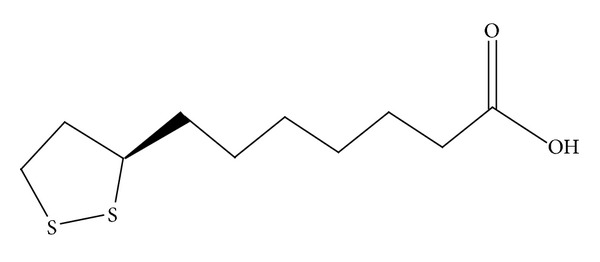
The structure of ALA.

Alpha-lipoic acid (ALA), a thiol compound (Figure 1), occurring naturally in human diet food, animal tissues, and plants has been reported to exhibit antioxidant [10] and anti-inflammatory activities through inhibition of the release of proinflammatory cytokines, NO and ROS caused by LPS in macrophages and Kupffer cells [11]. Recently, it has been demonstrated that ALA can upregulate HO-1 expression in human monocytic cells [12], suggesting that HO-1 may involve the actions of ALA. However, there is no information available about whether ALA exerts a protective effect in ALI caused by LPS. In the present study, we demonstrated for the first time that induction of HO-1 may contribute to the ALA-mediated protection against LPS-induced ALI.

## 2. Materials and Methods

### 2.1. Materials

Lipopolysaccharide (*Escherichia coli*, serotype 0127:B8) were purchased from Sigma-Aldrich (Louis, MO, USA). Polyclonal anti-HO-1 and monoclonal anti-phospho-NF-*κ*B p65 (Ser536) antibodies were purchased from Cell Signaling (Danvers, MA, USA). Mouse polyclonal anti-iNOS and horseradish peroxidase-conjugated anti-rabbit immunoglobulin (IgG) antibodies were from Abcam (Cambridge, UK). Mouse polyclonal anti-*β*-actin, anti-nitrotyrosine, and fluorescein isothiocyanate- (FITC-) conjugated anti-rabbities IgG antibodies were obtained from Santa Cruz (Santa Cruz, CA, USA). Rabbit monoclonal anti-phospho-Nrf2 (L593) antibody was purchased from Bioworld (Louis, MN, USA). The ELISA kits of TNF-*α*, IL-1*β*, IL-6, and CINC-3 were purchased from R&D systems (Abingdon, UK). The racemic RS (+/−)-ALA (Sigma-Aldrich, St. Louis, MO, USA) was dissolved in an aqueous alkaline solution (1 N NaOH) followed by the addition of Tyrode solution, and the pH was neutralized with 1 N HCl. 

### 2.2. Animals

Male Sprague-Dawley rats weighing 200–230 g were used for this study. All experimental procedures were conducted in accordance with the Guiding Principle in the Care and Use of Animals and approved by the Institutional Animal Care and Use Committee of National Defense Medical Center (Taipei, Taiwan, ROC).

### 2.3. LPS-Induced ALI Model and Experimental Design

Four groups of Sprague-Dawley rats were used: (1) control rats were administrated with an intratracheal (i.t.) instillation of phosphate buffered saline (PBS); (2) LPS-treated group received an instillation of LPS (5 mg/kg body weight, i.t.) in 0.2 mL PBS; (3) LPS+ALA-treated group injected with ALA (100 mg/kg body weight) intraperitoneally (i.p.) 1 h before (pretreatment) or 3 h after (posttreatment) instillation of LPS (5 mg/kg body weight, i.t.); (4) LPS+ALA+SnPP-treated group injected with SnPP (50 mg/kg body weight, i.p.), a selective inhibitor of HO-1, 2 h before and ALA (100 mg/kg body weight, i.p.) 1 h before instillation of LPS (5 mg/kg body weight, i.t.). Rats were euthanized at 6 h after LPS instillation, and the samples were collected for further analysis. For intratracheal instillation, rats were anesthetized with a single intraperitoneal dose of ketamine (90 mg/kg body weight) and xylazine (7 mg/kg body weight), followed by administration of LPS intratracheally through a 24-gauge catheter as previously described [13]. 

### 2.4. Bronchoalveolar Lavage Fluid (BALF) Collection and Measurement of Cytokine, Cell Counts and Protein Concentration

At the end of experiments, the right lungs were ligated at the right main bronchus and the BALF was collected from left lungs through a tracheal cannula with 5 mL of autoclaved PBS. The collected BALF (4 mL) was centrifuged at 300 g for 10 min at 4°C, and the supernatants of BALF were stored at −70°C until use. The cell pellets were resuspended in PBS for total cell number counting by using a standard hemocytometer. The levels of tumor necrosis factor-*α* (TNF-*α*), interleukin-1*β* (IL-1*β*), interleukin-6 (IL-6), and cytokine inducible neutrophil chemoattractant-3 (CINC-3) in BALF were determined by enzyme-linked immunasorbent (ELISA) kits (R&D Systems, Abingdon, UK), respectively. The protein concentration in BALF supernatants was measured by the Bradford method using a Bio-Rad protein assay kit (Bio-Rad Laboratories, Hemel Hempstead, UK) with bovine serum albumin (Sigma) as a standard.

### 2.5. Myeloperoxidase (MPO) Activity

MPO activity, an indicator of PMN accumulation in lungs, was determined as previously described [14]. Lung tissues were homogenized and sonicated in 50 mM potassium phosphate (pH 6) containing 5 mM hexadecyltrimethylammonium bromide (Sigma-Aldrich, St. Louis, MO, USA) and subjected to three cycles of freezing and thawing followed by centrifugation at 14,000 g for 10 min at 4°C to obtain the supernatant. MPO activity was determined by combining 20 *μ*L tissue supernatant with 40 *μ*L assay buffer containing 0.167 mg/mL O-dianisidine dihydrochloride (Sigma-Aldrich, St. Louis, MO, USA) and 0.0005% hydrogen peroxide. The MPO activity was measured dynamically using a spectrophotometer (Scinco UV-2120, Seoul, Korea) at 460 nm over 3 min. The MPO activity was calculated as the change in absorbance (ΔA) at 460 nm over 1 min and expressed as ΔA_460_/min/g protein.

### 2.6. Lung Wet/Dry Weight Ratio

After euthanasia of rats, the right lungs were immediately weighed to get the wet weight and then placed in an oven at 60°C for 48 h and weighed to obtain the dry weight. The ratio of the wet lung to the dry lung was calculated to assess tissue edema. 

### 2.7. Histological Examination

The lung tissues were fixed with 4% (w/v) paraformaldehyde, dehydrated in graded ethanol, and embedded in paraffin, and tissue sections (4–6 *μ*m thick) were stained with hematoxylin and eosin (H&E) under light microscope. To determine the score of the lung injury, the histological images were evaluated by an investigator who was initially blinded to these research groups. The lung injury was scored according to the following principle: (1) alveolar congestion, (2) hemorrhage, (3) infiltration or aggregation of neutrophils in the airspace or vessel wall, and (4) thickness of the alveolar wall/hyaline membrane formation. Each item was graded according to a four-point scale from 0 to 3 as follows: 0 = no damage, 1 = mild damage, 2 = moderate damage, and 3 = severe damage [15]. 

### 2.8. Measurement of O_2_^−^ Production in Lung

The freshly harvested right lung was cut into pieces of 5 × 5 mm and incubated with oxygenated Krebs-HEPES buffer for 5–10 min. Then, the pieces of lung tissue were transferred to 96-well microplates. These microplates containing 1.25 mM lucigenin in 200 *μ*L Krebs-HEPES buffer were placed into a microplate luminometer (Microlumat LB96V, Berthold, Germany). Counts of chemiluminescence signal from lung were obtained at 1 min intervals at room temperature. The counts of plates containing all components without lung tissue were regarded as background, and the blank value was subtracted from the chemiluminescence signals obtained from the lung samples. All lung tissues were weighed after drying at 60°C for 48 h. The results were expressed as reactive luminescence units (RLU) per 1 min per milligram dry weight (RLU/min/mg dry weight). In addition, the lung sections were incubated with fluorescent oxidation product of dihydroethidium (DHE, 10 *μ*M) for 15 min to monitor superoxide production. After extensive washings with TBS, the coverslips were mounted onto the glass slides and the fluorescence images were photographed with a fluorescence microscope (Leica DMI6000B, Wetzlar, Germany).

The image analysis was performed with Metamorph NX (Molecular Devices) to quantify the fluorescence intensity.

### 2.9. Measurement of Nitrite/Nitrate (NO_X_) Level in BALF

The amount of NO was examined by measuring the intermediate and end products, NO_x_. The BALF was added with two volumes of ethanol at 20°C for 2 h to precipitate protein. Then, the NO_x_ in samples were reduced to NO by adding a reducing agent (0.8% VCl_3_ in 1 N HCl). Then, the NO was determined by a Sievers Nitric Oxide Analyzer (Sievers 280 NOA, Sievers, Boulder, CO, USA). Nitrate concentration was calculated by comparison with a standard solution of sodium nitrate.

### 2.10. Immunohistochemistry Staining for Nitrotyrosine

The lung sections were deparaffinized, dehydrated, and immersed in 10 mM sodium citrate buffer for 5 min at 100°C. After blocking with 5% bovine serum albumin at room temperature for 20 min, the slides were incubated at 4°C with a polyclonal nitrotyrosine primary antibody (1 : 50 dilution; Santa Cruz Biotechnology, CA, USA) overnight, followed by addition of a goat anti-rabbit immunoglobulin horseradish peroxidase-conjugated secondary antibody (1 : 50; Abcam, Cambridge, UK) for 1 h at room temperature. The presence of nitrotyrosine was indicated by the brown peroxidase reaction product and photographed with light micrographs (Leica DMI6000B, Wetzlar, Germany). The quantitative analysis of immunostaining was performed using ImageJ Plugins to calculate the area of immunoreactions with nitrotyrosine.

### 2.11. Assay of NF-*κ*B and Nrf2 Translocation

The lung tissue slices mounted on the coverslips were incubated with rabbit monoclonal phospho-NF-*κ*B p65 (Ser536) or anti-phospho-Nrf2 (L593) primary antibody overnight at 4°C diluted 1 : 100 in 1% BSA in TBS. After washing, coverslips were incubated with a fluorescein-isothiocyanate (FITC-) conjugated secondary antibody diluted 1 : 100 with 1% BSA in TBS for 1 h. In addition, the coverslips were stained with Hoechst 33258 dye in the nuclei. After extensive washings with TBS, the coverslips were mounted onto the glass slides and photographed with a fluorescence microscope (Leica DMI6000B, Wetzlar, Germany). The analysis of images was performed with Metamorph NX (Molecular Devices) to quantify the nuclear phospho-NF-*κ*B p65 or phospho-Nrf2 staining and proportion of cells demonstrating positive colocalization of DAPI/phospho-NF-*κ*B p65 or phospho-Nrf2 staining.

### 2.12. Western Blot Analysis

Lung tissues were homogenized in RIPA lysis buffer (50 mM Tris-HCl, pH 7.4, 150 mM NaCl, 0.25% deoxycholic acid, 1% NP-40, and 1 mM EDTA) containing EDTA-free protease inhibitor cocktail (Thermo Scientific, USA). The proteins were extracted at 4°C for 30 min and centrifuged twice at 10,000 g for 10 min at 4°C, and protein concentrations were determined by using Bio-Rad protein assay kit. The samples (100 *μ*g protein/lane) were loaded and separated on 10% sodium-dodecyl-sulfate-(SDS-) polyacrylamide (PAGE). The separated proteins were transferred to polyvinylidene fluoride membranes (Immobilon-P; Millipore, Bedford, MA, USA) and blocked with 5% skim milk in TBST (20 mM Tris-base, pH 7.5, 500 mM NaCl, and 0.1% Tween 20, v/v) for 1 h. After blocking, the membranes were incubated overnight at 4°C with specific primary antibodies against iNOS (1 : 1000 dilution), HO-1 (1 : 500 dilution), or *β*-actin (1 : 5000) in 5% skim milk. After additional washes, the membranes were incubated with a goat anti-rabbit immunoglobulin horseradish peroxidase-coupled secondary antibody at a dilution of 10,000 in 5% skim milk for 1 h. The immune-reactive bands were visible after development with a chemiluminescence (ECL) reagent (Amersham International Plc., Buckinghamshire, UK) and visualized on X-ray films. The bands were further quantified by densitometry and normalized with respective *β*-actin.

### 2.13. Statistical Analysis

The experimental data were expressed as the mean ± SEM. One-way ANOVA with post hoc Bonferroni test was used for statistical analysis. Results were considered significantly different at a value of *P* < 0.05.

## 3. Results

### 3.1. Effect of ALA on HO-1 Expression and Nrf2 Activation

**Figure 2:**
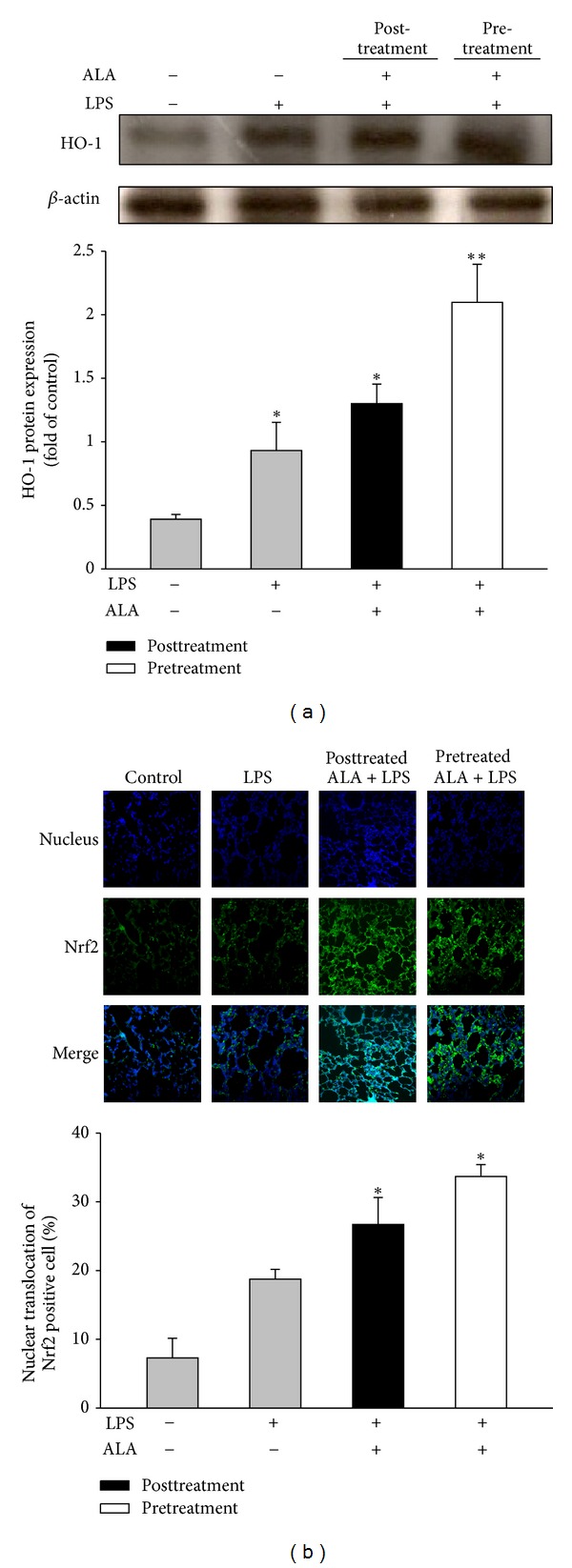
Effect of ALA on HO-1 expression and Nrf2 translocation in lungs instilled by LPS. Rats were treated with ALA (100 mg/kg, i.p.) 1 h before or 3 h after intratracheal instillation of LPS (5 mg/kg) for 6 h. Lungs were harvested for HO-1 protein expression by Western blot (a), and Nrf2 translocation assays with immunofluorescence staining (b). Rats received with vehicle (i.p.) and intratracheal instillation of PBS alone acted as control group. Data were represented as means ± SEM (*n* = 5). *P < 0.05; **P < 0.01 versus LPS-instilled alone group.

The protein expression of HO-1 in lung tissues was significantly higher in LPS-instilled group than that in control group. Pretreatment or posttreatment with ALA (100 mg/kg, i.p.) further enhanced the HO-1 expression (Figure 2(a)), which was in accord with the elevation of nuclear translocation of Nrf2 (Figure 2(b)). These results suggest that ALA-mediated induction of HO-1 may be through Nrf2 activation.

### 3.2. Effect of ALA on Leukocyte Accumulation in BALF and Lung MPO Activity

**Figure 3:**
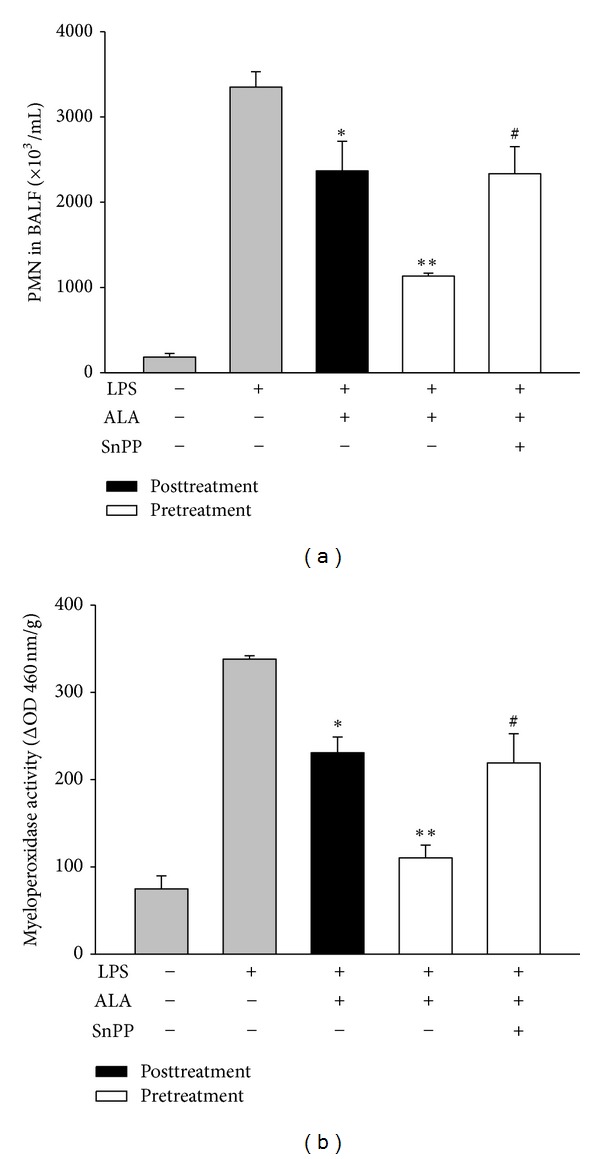
Effect of SnPP on the inhibition of ALA on LPS-induced neutrophil accumulation in BALF and lung MPO activity. Rats were intraperitoneally injected with ALA 1 h before or 3 h after intratracheal instillation of LPS (5 mg/kg). To investigate the role of HO-1, SnPP (50 mg/kg, i.p.) was administered 2 h and ALA 1 h before LPS instillation. The total leukocyte counts in BALF (a) and lung MPO activity (b) were measured at 6 h after LPS instillation. Data were presented as means ± SEM (*n* = 5). *P < 0.05; **P < 0.01 versus LPS-instilled alone group; ^#^ *P* < 0.05 versus LPS+ALA group.

LPS instillation markedly increased the number of total leukocytes in BALF and lung MPO activity. However, pretreatment or posttreatment with ALA significantly reduced the increase caused by LPS. To examine the role of HO-1, the SnPP (50 mg/kg, i.p.), an HO-1 inhibitor, was added, and our data showed that blocking HO-1 activity significantly reversed the inhibition of pretreatment with ALA on LPS-induced leukocyte accumulation and lung MPO activity (Figures 3(a) and 3(b)).

### 3.3. Effect of SnPP on ALA-Mediated Reduction of Protein Level in BALF and Lung Edema

**Figure 4:**
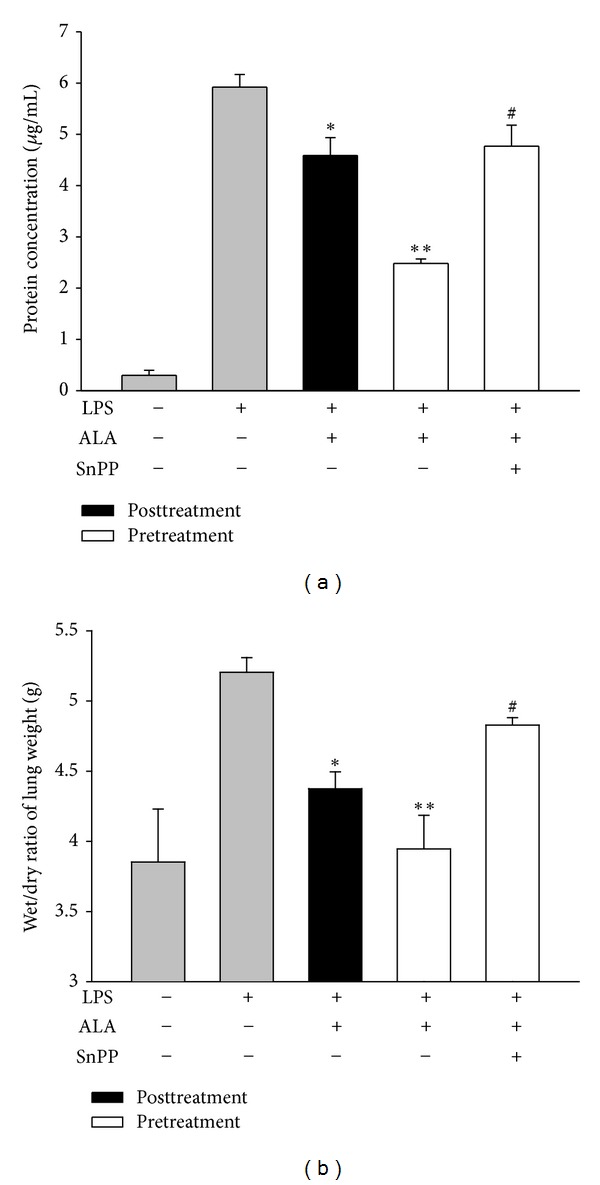
Effects of SnPP on the attenuation of ALA on LPS-induced protein accumulation in BALF and lung wet/dry weight ratio. Rats were intraperitoneally injected with ALA 1 h before or 3 h after intratracheal instillation of LPS (5 mg/kg). In some rats, SnPP was administered 2 h and ALA 1 h before LPS instillation. The protein level in BALF (a) and pulmonary edema determined by the wet/dry weight ratio (b) were measured at 6 h after LPS instillation. Data were presented as means ± SEM (*n* = 5). *P < 0.05; **P < 0.01 versus LPS-instilled alone group; ^#^ *P* < 0.05 versus LPS+ALA group.

Compared with control group, intratracheal instillation of LPS resulted in a significant increase in protein level in BALF and lung edema evidenced by elevation of lung wet/dry weight ratio. Pretreatment or posttreatment with ALA significantly attenuated the increase of lung edema caused by LPS. However, cotreatment with SnPP markedly reversed pretreatment with ALA-mediated reduction of protein leakage and lung edema (Figures 4(a) and 4(b)). 

### 3.4. Effect of SnPP on ALA-Mediated Improvement of Lung Histopathological Changes

**Figure 5:**
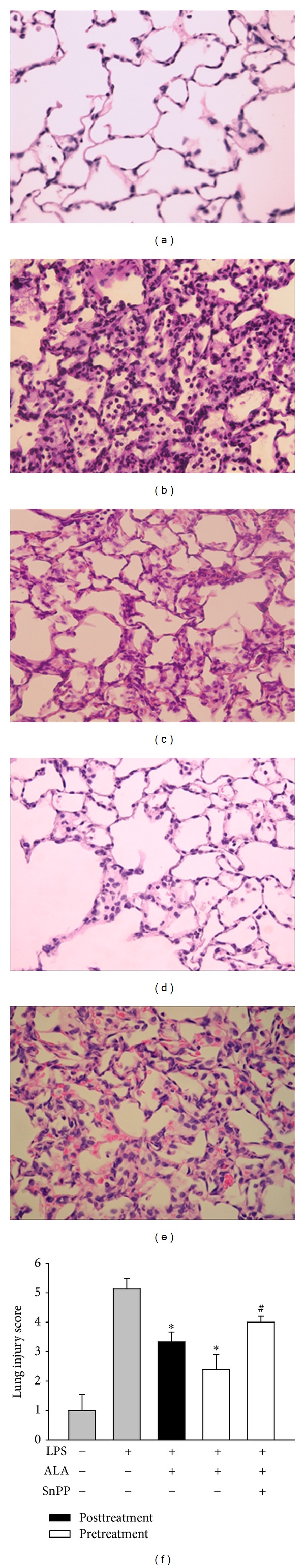
Effect of SnPP on the improvement of ALA on LPS-induced lung histopathological changes. Rats were intraperitoneally injected with ALA 1 h before or 3 h after intratracheal instillation of LPS. In some rats, SnPP was administered 2 h and ALA 1 h before LPS instillation. The histopathological assays (200× magnification) in lungs were performed at 6 h after LPS instillation in control group (a), LPS-instilled alone group (b), ALA (posttreatment)+LPS group (c), ALA (pretreatment)+LPS group (d), SnPP+ALA (pretreatment)+LPS group (e). The lung injury score was also determined (f). Data were represented as means ± SEM (*n* = 5). *P < 0.05 versus LPS-instilled alone group; ^#^ *P* < 0.05 versus LPS+ALA group.

After LPS challenge, the morphological examination showed that there were several histopathological alterations including cell structure destructed, lung edema, alveolar wall thickening, and neutrophil infiltration observed in the lungs. Pretreatment or posttreatment with ALA remarkably improved LPS-induced pathological symptoms in lungs, which were markedly reduced by blocking HO-1 activity with SnPP evidenced by the score of the lungs (Figure 5). These findings indicate that ALA exerts a preventive and therapeutic effect in LPS-induced ALI.

### 3.5. Effect of SnPP on ALA-Mediated Inhibition of Cytokine Level in BALF

**Figure 6:**
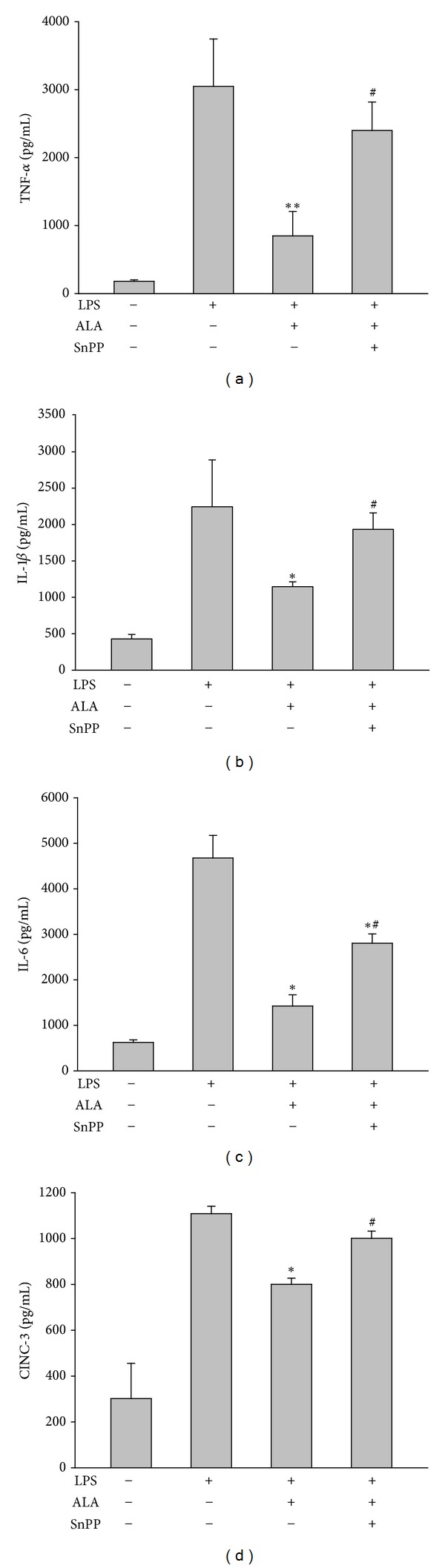
Effect of SnPP on the inhibition of ALA on LPS-induced proinflammatory cytokine and CINC-3 production in BALF. Rats were pretreated with ALA 1 h before intratracheal instillation of LPS for 6 h. In some rats, SnPP was administered 2 h and ALA 1 h before LPS instillation. Data were presented as means ± SEM (*n* = 5). *P < 0.05; **P < 0.01 versus LPS-instilled alone group; ^#^ *P* < 0.05 versus LPS+ALA group.

It is known that proinflammatory cytokines including TNF-*α*, IL-1*β*, IL-6, and chemokine such as CINC-3 play a key role in the pathogenesis of ALI. We found that the levels of inflammatory mediators in BALF were markedly increased in rats that received LPS administration compared with control group. The augmentation of these inflammatory mediators induced by LPS was significantly inhibited by pretreatment with ALA, but cotreatment with SnPP remarkably attenuated the inhibition (Figure 6).

### 3.6. Effect of SnPP on ALA-Mediated Suppression of Superoxide Production in Lungs

**Figure 7:**
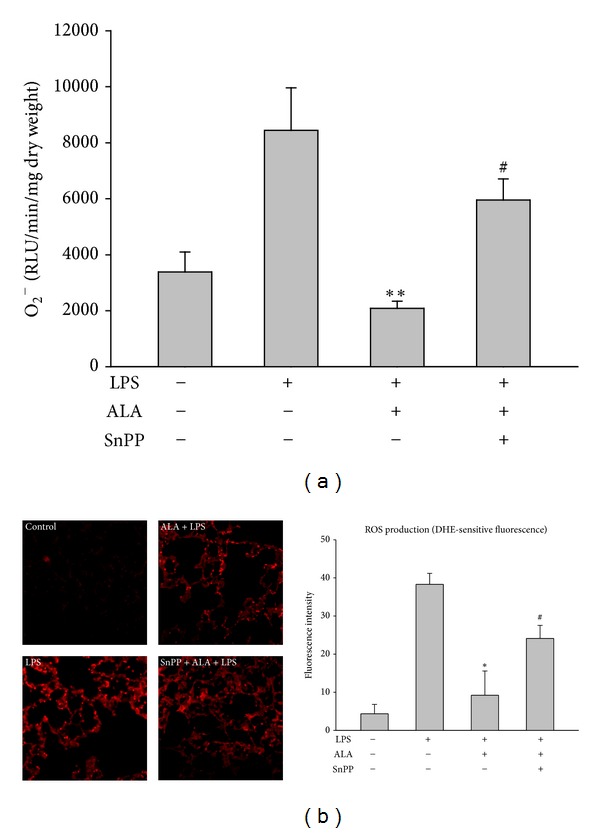
Effect of SnPP on the suppression of ALA on LPS-induced O_2_^−^ formation in lungs. Rats were pretreated with ALA 1 h before intratracheal instillation of LPS for 6 h. In some rats, SnPP was administered 2 h and ALA 1 h before LPS instillation. The lung O_2_^−^ formation was measured by lucigenin chemiluminescence (a) and DHE immunofluorescent staining (red) (b). Data were represented as means ± SEM (*n* = 5). *P < 0.05 versus LPS-instilled alone group; ^#^ *P* < 0.05 versus LPS+ALA group.

Pretreatment with ALA strongly inhibited LPS-induced O_2_^−^ production in lungs determined by lucigenin chemiluminescence (Figure 7(a)) and DHE fluorescence methods (Figure 7(b)). However, the inhibition by ALA was markedly abolished by SnPP, suggesting that the antioxidant effect of ALA is associated with the action of HO-1. 

### 3.7. Effect of SnPP on ALA-Mediated Reduction of NO_x_ Content in BALF, Expression of iNOS and Nitrotyrosine in Lungs

**Figure 8:**
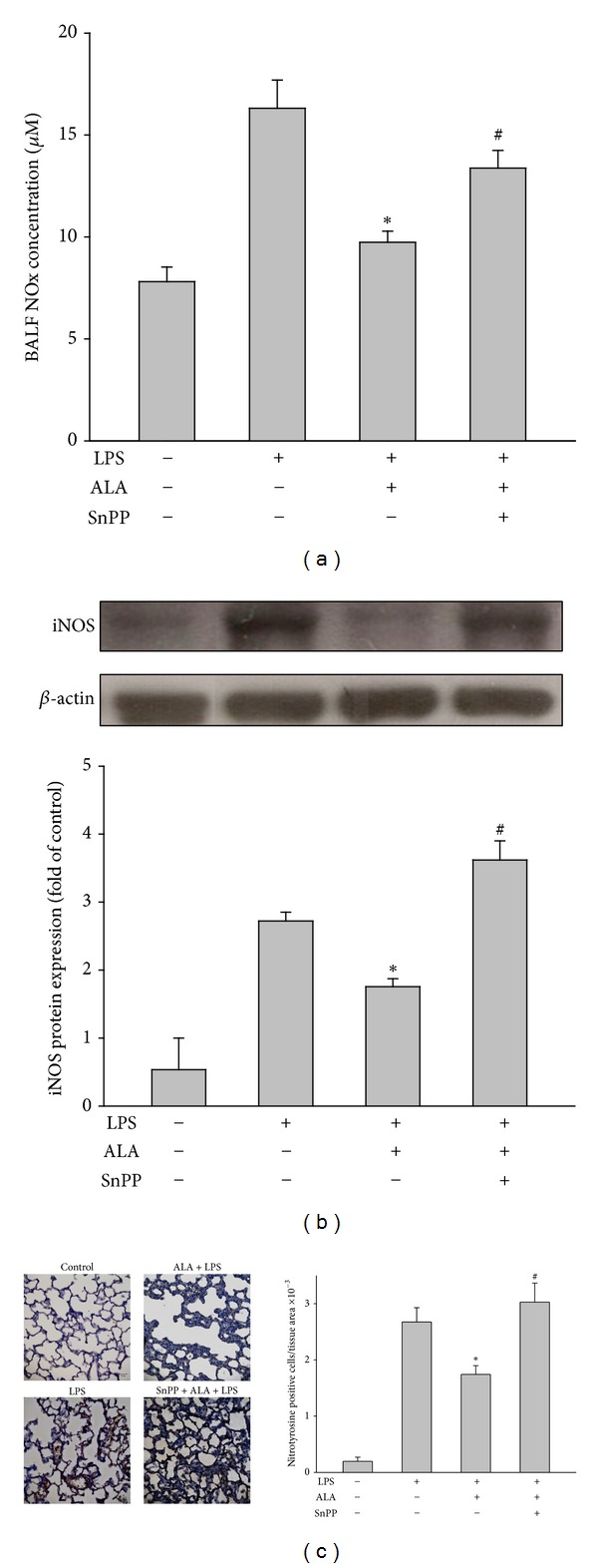
Effects of SnPP on the inhibition of ALA on LPS-induced NO_x_ level in BALF, iNOS and nitrotyrosine expression in lung tissues. Rats were pretreated with ALA 1 h before intratracheal instillation of LPS for 6 h. In some rats, SnPP was administered 2 h and ALA 1 h before LPS instillation. The NO_x_ level in BALF (a), iNOS (b) and nitrotyrosine expression (c) in lungs were measured. Data were represented as means ± SEM (*n* = 5). *P < 0.05 versus LPS-instilled alone group; ^#^ *P* < 0.05 versus LPS+ALA group.

The levels of NO_x_ in BALF, iNOS and nitrotyrosine expression in lungs were significantly increased in LPS-instilled alone group compared with those in control group. Pretreatment with ALA significantly inhibited the increase caused by LPS, which was significantly attenuated by cotreatment with SnPP (Figure 8).

### 3.8. Effect of SnPP on ALA-Mediated Suppression of NF-*κ*B Activation

**Figure 9:**
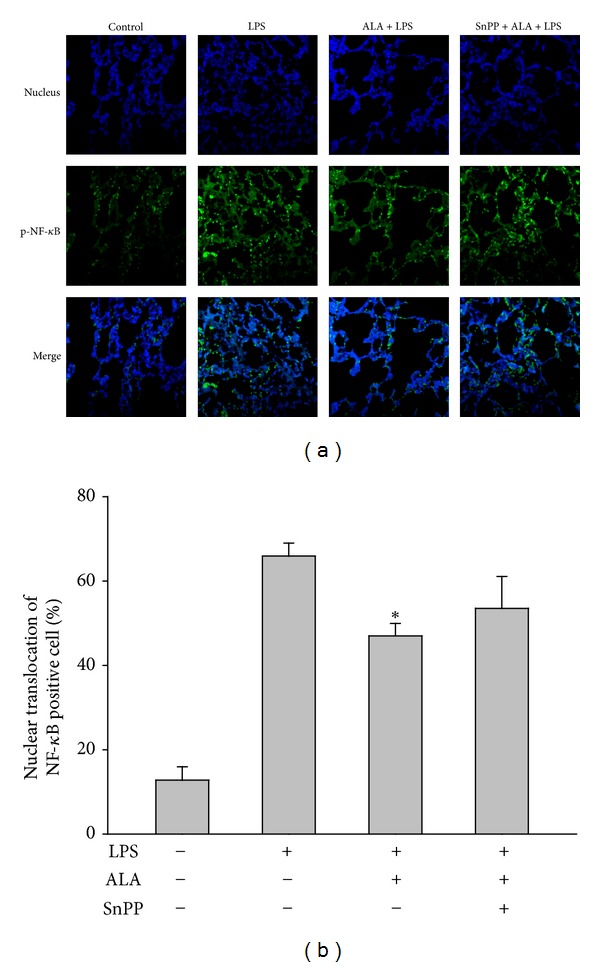
Effect of SnPP on the reduction of ALA on LPS-induced NF-*κ*B activation in lungs. Rats were pretreated with ALA 1 h before intratracheal instillation of LPS for 6 h. In some rats, SnPP was administered 2 h and ALA 1 h before LPS instillation. The expression of phospho-NF*κ*B-p65 in nucleus of lungs with immunofluorescence stains was determined (a) and semiquantified (b) at 6 h after LPS instillation. Data were represented as means ± SEM (*n* = 5). *P < 0.05 versus LPS-instilled alone group.

Activation of NF-*κ*B is known to be critical for modulating LPS-induced inflammatory gene expression [16]. Immunofluorescent staining assay of lung tissues illustrated that there was increased nuclear translocation of phospho-p65NF-*κ*B in LPS-instilled alone rats compared with that of control group. Pretreatment with ALA significantly inhibited LPS-induced NF-*κ*B translocation, which was reduced by coadministration of SnPP (Figure 9). Therefore, ALA-mediated suppression of NF-*κ*B activation and subsequent inflammatory gene expression may be modulated by HO-1. Moreover, treatment with ALA or SnPP itself did not significantly affect these above mediators measured in this study.

## 4. Discussion

In the present study, we demonstrate for the first time that pretreatment or posttreatment with ALA significantly inhibited LPS-induced histological changes and inflammatory responses of ALI. Furthermore, there is evidence supporting our hypothesis that the protection of ALA against LPS-induced ALI is mediated by HO-1 induction. Firstly, ALA significantly enhanced HO-1 protein expression via Nrf2 activation in lungs exposed to LPS. Secondly, blocking HO-1 activity with SnPP dramatically attenuated ALA-mediated mitigation of histopathologic symptoms and inhibition of various inflammatory mediator formation and gene expression in lungs challenged by LPS. Taken together, these findings, we conclude that the protective effect of ALA in LPS-induced ALI is, at least in part, mediated by HO-1.

It has been reported that the Nrf2-modulated HO-1 induction is an important adaptive response to enhance cellular resistance to inflammatory insults [17]. Under normal condition, Nrf2 is sequestered in the cytoplasm by association with the repressor keap1. Upon exposure to stimuli, Nrf2 dissociates from keap1 and translocates into the nucleus leading to binding to antioxidant response element (ARE) in the promoter region of stress-inducible genes including HO-1 [18]. Importantly, we found that treatment with ALA increased Nrf2 translocation into nucleus, which was consistent with the induction of HO-1 protein expression in lungs instilled by LPS, suggesting that ALA-induced HO-1 expression may be due to Nrf2 activation. The HO-1-derived products of heme catabolism such as CO and biliverdin have been reported to exert a cytoprotective activity against oxidative and inflammatory insults in the pulmonary diseases including ALI [19]. Previous studies have indicated that the anti-inflammatory activity of HO-1 is mediated by regulation of immune-mediated inflammation, suppression of proinflammatory cytokine, chemokine, ROS formation, and iNOS expression through attenuation of NF-*κ*B activation in several inflammatory diseases [20, 21]. To investigate the role of HO-1 in the protective effect of ALA, SnPP was added to evaluate the alterations of these anti-inflammatory activities of ALA. 

Data from this study demonstrated that LPS-induced pulmonary pathological features of ALI including alveolitis, leukocytes infiltration, increase of alveolar wall thickness, protein leakage, and edema could be significantly attenuated by pretreatment or posttreatment with ALA. However, the protective effect of ALA was markedly diminished by cotreatment with SnPP, an inhibitor of HO-1, suggesting that the preventive and therapeutic effect of ALA is associated with HO-1-dependent processes. Accumulation of neutrophils in lung tissues has been closely correlated with the poor prognosis in septic ALI, and inhibiting recruitment of neutrophils mitigates LPS-induced lung injury [22]. ALA is known to exert anti-inflammatory and tissue-protective effects [23]. Here, we further confirmed that ALA markedly attenuated the elevation of CINC-3, a chemotactic cytokine, release, neutrophil accumulation in BALF and MPO activity, a marker of neutrophil influx into tissue, in lungs instilled by LPS. Furthermore, the increased level of proinflammatory cytokines, including TNF-*α*, IL-1*β*, and IL-6, in BALF secreted by activated alveolar inflammatory cells including neutrophils plays a critical role in causing ALI,and has a close association with the worst outcome in ALI and septic patients [24, 25]. Similarly, the LPS-induced elevation of proinflammatory cytokine level in BALF was significantly inhibited by ALA. However, cotreatment with SnPP markedly abolished the inhibitory effects of ALA on neutrophilia and proinflammatory cytokine formation in lungs challenged by LPS, suggesting that the anti-inflammatory activity of ALA may be through a mechanism that involves the actions of HO-1.

Exposure to LPS, the bursts of ROS released from immune cells and other cells located in the airways and circulation can induce inflammatory responses, subsequently causing lung injury [26]. Consistent with previous finding that HO-1 could attenuate ROS formation via inhibition of NADPH oxidase activity through reduction of heme availability [27], ALA-mediated suppression of LPS-induced O_2_^−^ formation in lungs was markedly abolished by pretreatment with SnPP. These results suggest that HO-1 involves the antioxidant activity of ALA. 

Another important mediator, NO, synthesized by the enzyme nitric oxide synthase (NOS), modulates several physiological functions. However, the inducible-nitric-oxide-synthase-(iNOS-) derived NO overproduction in response to LPS, proinflammatory cytokines, and ROS has been closely linked to the pathogenesis of LPS-induced ALI [28]. Clinical observations showed that pulmonary iNOS expression and level of NO_x_, stable breakdown by-products of NO, in BALF of patients with ARDS are much higher than those in normal subjects [29]. Moreover, in a murine model of LPS-induced ALI, it has been reported that the dysfunction of alveolar-capillary barrier and pulmonary edema are dependent on the increase of iNOS activity of alveolar inflammatory cells [28]. Additionally, the overproduction of NO can rapidly interact with O_2_^−^ to generate peroxynitrite, a strong oxidizing and cytotoxic product, leading to great damage to cells and tissues. Therefore, inhibition of iNOS/NO-related cytotoxicity is a potential treatment strategy in LPS-induced ALI. As expected, administration of ALA significantly inhibited LPS-induced iNOS expression and NO_x_ formation accompanied by a marked reduction of nitrotyrosine expression in lungs, but the inhibition was remarkably reversed by cotreatment with SnPP. Therefore, it is likely that HO-1-mediated attenuation of iNOS/NO pathway and ROS production may provide an important mechanism contributing to the protective effect of ALA in LPS-induced ALI. 

In quiescent cells, the NF-*κ*B is present as homodimeric or heterodimeric complexes of p50 and p65 subunits in cytoplasm and is bound to an inhibitory molecule, I*κ*B-*α*. Upon activation by LPS, I*κ*B-*α* is rapidly phosphorylated leading to a dissociation of I*κ*B-*α*/NF-*κ*B complex. Then, NF-*κ*B translocates into the nucleus and induces several inflammatory gene expression including iNOS and proinflammatory cytokines as well as ROS formation [16]. A lot of evidence indicates that LPS-induced NF-*κ*B activation in lungs results in pulmonary neutrophilia, epithelial permeability, and several inflammatory mediator formation [26]. Therefore, NF-*κ*B has been believed to be an important intracellular target for the early detection, prevention, and treatment of lung injury. Importantly, our data showed that ALA significantly suppressed LPS-induced translocation of p65NF-*κ*B into nucleus of lungs, but the inhibition was reduced by blocking HO-1 activity, indicating that ALA-mediated NF-*κ*B inactivation is also regulated by HO-1. Our finding is similar to previous studies indicating that the overexpression of HO-1 or treatment with CO, an end product of HO-1, significantly inhibits LPS-induced iNOS expression and NO production in macrophages via suppression of NF-*κ*B activation [21]. Although, the true mechanisms by which HO-1 exerts the anti-inflammatory activity are not understood well, limiting the proinflammatory free heme availability and producing the anti-inflammatory compounds such as biliverdin/bilirubin and CO may play a major role [30–32]. In conclusion, we demonstrated for the first time that ALA exhibits a protective effect in LPS-induced ALI, and the underlying mechanism may involve HO-1 induction, subsequently suppressing NF-*κ*B-mediated inflammatory responses.
